# A mixed-method evaluation of the adoption and implementation of the College Alcohol Intervention Matrix among prevention experts: a study protocol

**DOI:** 10.1186/s43058-021-00249-z

**Published:** 2021-12-20

**Authors:** Ashley C. Helle, Kenneth J. Sher, Joan Masters, Karla Washington, Kristin M. Hawley

**Affiliations:** 1 Department of Psychological Sciences, University of Missouri, 200 South 7th Street, Columbia, MO 65211 USA; 2 Department of Wellness, Student Affairs, University of Missouri, G202 MU Student Center, Columbia, MO 65211 USA; 3 Division of Palliative Medicine, School of Medicine, Washington University, 660 S. Euclid Ave, St. Louis, MO 63110 USA

**Keywords:** College prevention, Substance use prevention, Implementation, Adoption

## Abstract

**Background:**

Risky drinking among college campuses has been a long-standing concern and there have been dedicated efforts to develop evidence-based prevention and treatment strategies (EBSs) to decrease alcohol use and increase healthy behaviors among college students. Further, the College Alcohol Intervention Matrix (CollegeAIM) was developed as a tool with accompanying resources, to assist institutions of higher education in selecting EBSs that are appropriate and a good fit for their campuses. However, the CollegeAIM tool and selection of prevention strategies from stakeholders’ perspectives has yet to be evaluated. This study protocol describes the methodology for a research project evaluating CollegeAIM from an implementation science perspective using the Exploration, Preparation, Implementation, and Sustainment framework.

**Methods:**

The aims of this study will be accomplished with a mixed-method design comprised of reviews of strategic planning documents, quantitative surveys and interviews with prevention experts, and focus groups to identify key components of a decision-support program for prevention experts to support the use of CollegeAIM. Participants are members of the multi-site Missouri Partners in Prevention coalition to reduce risky substance use on college campuses across the state.

**Discussion:**

The results of this study will provide key information to support the development of additional supportive tools for campuses that can improve their selection and implementation of EBSs that fit the needs of their respective campuses. This work is important to further advance the implementation and sustainment of extant EBSs for risky college alcohol use.

Contributions to the literature

- College students are a group at high-risk for alcohol-related risks and consequences and universities are tasked with alcohol prevention activities.
- There are a number of evidence-based strategies for alcohol prevention, which are listed in the College Alcohol Intervention Matrix tool (CollegeAIM).
- Studies have yet to evaluate the adoption and implementation of the CollegeAIM tool and gather the perspectives of prevention specialists.
- This study will evaluate the use of CollegeAIM in order to support the development of supportive tools for campuses to improve alcohol prevention and support prevention specialists.

## Introduction

### Background

Risky drinking and substance use among college students has been a longstanding concern. Serious negative outcomes and consequences (e.g., unintentional injuries, sexual assault, heavy consumption, poisoning, and death) are consistently associated with risky use [1–4]. Although we are seeing decreases in some domains of risky substance use (e.g., binge drinking rates are on the decline [5, 6], there are still areas in need of improvement (e.g., risky cannabis use, supporting students in recovery). There are also burgeoning concerns, such as the prevalence and consequences of concurrent and simultaneous use of alcohol with other substances [7, 8] and high intensity drinking, which is defined as drinking significantly more standard drinks than a binge, in a single setting (i.e., 12+ drinks for women, 15+ drinks for men) [9, 10], just to name a few.

For the past 20+ years, there has been a concerted effort to address alcohol use at the college level, including the “A Call to Action” initiative and report [11], which involved bringing together the expertise of alcohol researchers, members of higher education, and students, resulting in a report outlining evidence-based strategies (EBSs) to combat risky drinking. Later, NIAAA’s Rapid Response initiative was employed, which connected teams of alcohol researchers with higher education institutions, and resulted in a series of projects that provided information on prevention and treatment strategies for college student drinking [12]. About 15 years after the initial call to action initiative, NIAAA brought together teams of experts to develop the NIAAA College Alcohol Intervention Matrix (CollegeAIM) [13, 14], a freely-available tool designed for staff at higher education institutions who are wanting to reduce risky alcohol use on their campuses. The CollegeAIM tool includes two matrices: one focuses on individual evidence-based strategies (EBSs) and the other on environmental EBSs. Within each matrix, the EBSs are organized by cost and effectiveness and include other key factors such as degree of barriers (e.g., high, moderate) to implementation. CollegeAIM was carefully crafted to help colleges and universities advance the implementation of EBSs for drinking among college students. However, the application of CollegeAIM has yet to be empirically investigated so the extent to which it actually supports selection and implementation of EBSs in college is unknown. Indeed, there is surprisingly little implementation-specific research focused on alcohol interventions for college students.

### Implementation science in college drinking

There is a wealth of research on the development and efficacy of EBSs for alcohol use among college students. From this empirical work, we know that evidence-based prevention and treatment approaches exist. However, realizing the public health impact of any EBS can and often is stalled in mental health and substance abuse fields, in the transition from the establishment of treatment efficacy to the actual uptake and full implementation of the intervention [15]. The decision to select and implement an EBS on a given campus is complex given the numerous contexts and stakeholders involved (e.g., administration, health educators, law enforcement), including the community in which the institution is embedded. The context and related complexity provides several points at which EBSs selection and implementation can be maximized *or* break down.

The current study seeks to address one section of this pipeline from the initial development of an evidence-based practice to full, effective implementation and sustainability: the selection and adoption of EBSs. Although few studies are applying an implementation lens to study prevention and treatment for alcohol on college campuses, there are many institutions and prevention coalitions devoting their applied efforts toward addressing risky drinking. Ideally, this process would be empirically examined in order to improve and streamline the process for prevention experts, to increase implementation, and to improve drinking outcomes for other institutions as well. Given that reduction in risky drinking and associated consequences is the ultimate goal, *selection* of acceptable, feasible, and effective strategies to address college drinking is a necessity.

Implementation science is a field uniquely equipped to address this process. Decades of intervention development and efficacy research and teams of experts have worked to develop a menu of EBS options to address risky college drinking (i.e., CollegeAIM). We can now investigate the initial steps taken by the stakeholders, including the selection, adoption, and implementation of EBSs with mixed-methods research and implementation science frameworks. With the wealth of research focused on efficacy of the interventions, now is the time to use implementation science approaches to determine how we can further accelerate strategies for risky drinking.

The Exploration, Preparation, Implementation, and Sustainment (EPIS) framework conceptualizes implementation as a “process” and considers inner and outer contextual factors, intervention characteristics, and bridging factors (e.g., prevention team-college partnerships) across the four stages of implementation [16]. The EPIS framework has been used in implementation studies across systems (e.g., public health, education) and health domains (e.g., substance use, HIV [17]). The EPIS framework was identified as the ideal lens by which to examine CollegeAIM adoption and implementation given the focus and versatility of the framework, along with key aspects related to bridging factors that are relevant to this research question. Studying implementation outcomes (e.g., appropriateness, feasibility) related to CollegeAIM and the selection of prevention and intervention strategies will provide clarity regarding the process by which institutions use the tool to select strategies to implement, and will point to key areas for the integration of support (e.g., provider training, resources, technical assistance) to improve the adoption and implementation process for college drinking EBSs.

### Current study and implementation context

#### Focus of the current study

This study will examine the use of the CollegeAIM tool as part of the selection and implementation of evidence-based strategies for risky drinking on college campuses. This project is important given the availability and vast amount of information contained in the CollegeAIM tool, but with no research related to whether and how it is used and implemented and/or if stakeholders find it effective. Specifically, this study focuses on stakeholders’ actions and perspectives: how are decisions made regarding which prevention and intervention strategies to employ on their campuses? Does CollegeAIM play a role? If so, how, and what are the barriers and facilitators to CollegeAIM use and selection of EBSs from a wide lens perspective (e.g., inner and outer context, features of CollegeAIM itself)? Ideally, the CollegeAIM matrix and accompanying resources (e.g., planning sheet) would serve as a toolkit to help institutions of higher education review and select EBSs that they find acceptable, appropriate, feasible, and sustainable for their respective campus and needs. However, we need to understand, from the stakeholders themselves, how these decisions are being made. This inquiry will ideally lead to improvement of adoption and implementation of EBSs—with the ultimate goal of positively impacting outcomes for students related to alcohol and other substance use.

#### Partnership and context

The aims of this study will be achieved through the collaboration between the research team and Missouri Partners in Prevention (PIP), a coalition comprised of higher education institutions across the state of Missouri, with a focus on substance use prevention and health and wellbeing among college students. The PIP director (co-author Masters) and central office PIP team staff have worked collaboratively with the research team to establish the study procedures. The stakeholders (i.e., primary participants in this study), include faculty members, members of administration, and staff members at the 23 PIP-institutions (colleges and universities) who are involved in the selection and implementation of substance-focused prevention and treatment efforts on their respective campuses. The member campuses have been involved with the PIP coalition prior to the study beginning and their membership in the coalition is not dependent on participating in this study—participation is distinct and completely voluntary. Working with this coalition will allow for gathering information about evidence-based strategy selection and implementation from a set of diverse campuses that vary in size, location, student body, and other demographic factors. Additionally, this partnership context in and of itself may be relevant to the adoption and implementation of prevention strategies (e.g., bridging factor in the EPIS model).

## Method

This research study has Institutional Review Board approval (IRB #2040543) at the University of Missouri. Any protocol modifications that occur will be submitted for approval to the IRB, prior to implementation.

### Study aims

This study uses a mixed-methods design to address three primary aims:

1. Evaluate the current use of CollegeAIM and define “effective” CollegeAIM use via administration of quantitative surveys, semi-structured interviews, and a strategic plan document review process. To accomplish this aim, we will evaluate stakeholders’ reports of how they select prevention strategies, how they use and perceive the CollegeAIM tool, as well as how CollegeAIM is used in conjunction with the annual strategic planning process at their respective institutions. Additionally, we will evaluate proximal outcomes of effective CollegeAIM use (i.e., do stakeholders believe CollegeAIM is useful in the selection of acceptable, appropriate, feasible, and sustainable EBSs that fit the needs of their institution).
2. Identify key determinants (i.e., barriers, facilitators) of effective CollegeAIM use and examine differences in determinants across institutions grouped by key characteristics (e.g., institution size, demographics, rural/urban designation). To accomplish this aim, we will administer quantitative surveys and conduct semi-structured interviews with key stakeholders to identify key determinants, which will be organized within the EPIS framework. Institution characteristics are identified via publicly available sources of higher education classifications (e.g., Carnegie classifications).
3. Develop an organizational decision-support program to enhance the effective application of CollegeAIM among prevention specialists on college campuses. This aim will harmonize information collected in prior aims to operationalize the process of CollegeAIM use and key determinants of use, which will then be used for the initial development of an organizational decision support program. After designing the initial components, we will elicit feedback from stakeholders via the use of focus groups. The focus groups will concentrate on stakeholder perspectives regarding the determinants that will be targeted in the organizational decision support program. This aim seeks to establish proof of concept for the new program that will be most useful for the stakeholders across a diverse set of institutions.

### Study phases and design

**Fig. 1:**
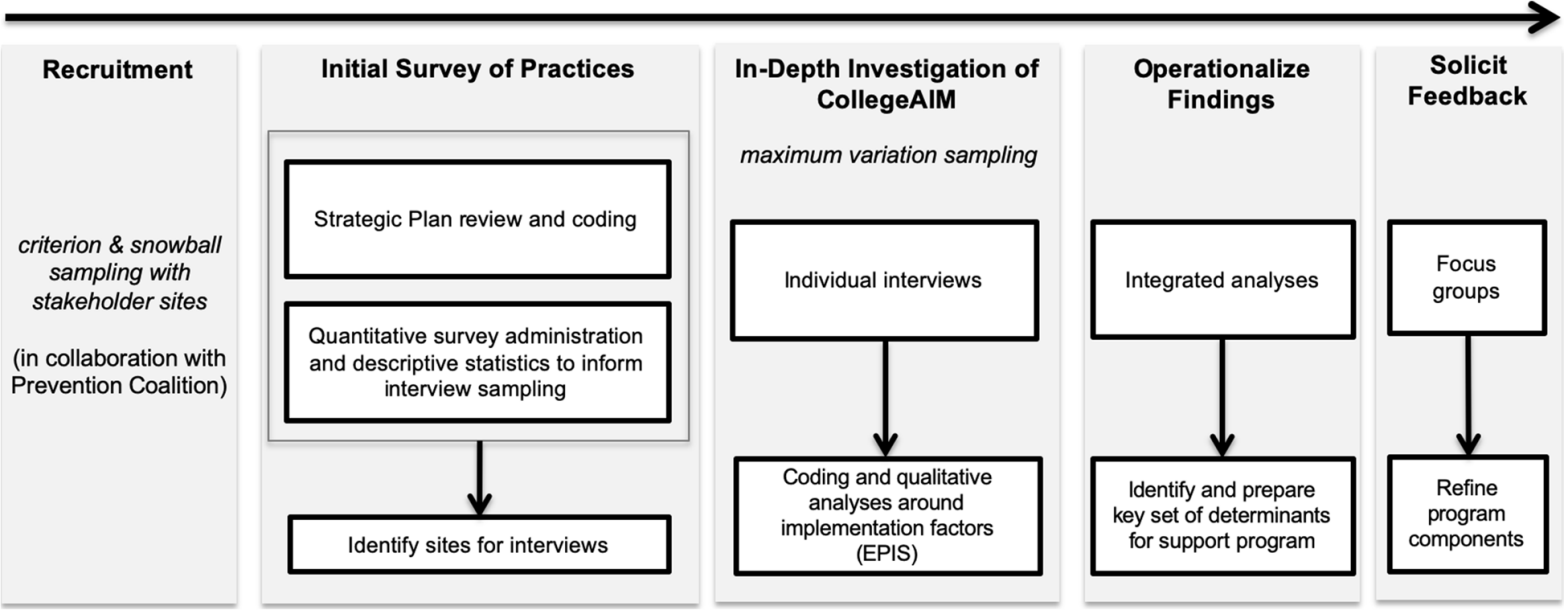
Study Procedures: Mixed-method design

A mixed-method design will guide this project (see Fig. 1). Qualitative and mixed-method approaches allow for in-depth investigations of stakeholder perspectives and “process,” which are key for implementation and getting a comprehensive understanding of whether and how CollegeAIM is currently used, as well as identifying areas in which additional support is needed to improve effectiveness [18, 19]. It should be noted that all data will be de-identified (i.e., individual participant identifiers  and campus names will be masked).

### Participants

Participants are stakeholders in higher education, specifically, higher education employees who are (1) affiliated with the prevention coalition, (2) have a role in the selection, adoption, and/or implementation of evidence-based strategies to address high risk drinking and/or substance use on their respective campuses, and (3) are a member of a PIP institution who has committed to the project collaboration. Members of the coalition may include counselors, psychologists, faculty members, healthcare professionals, members of residence life, conduct and/or law enforcement, and administration.

### Strategic plan review

Following informed consent from the primary campus contact, participating institutions will submit their annual strategic planning document for review. The strategic plans are completed by stakeholders each summer/fall for the upcoming school year. The plans include target/problem areas based on the prior year student assessment and outline planned strategies to address each area for the following academic year. The research team will extract data via double data-entry from the strategic plans regarding use of CollegeAIM, as well as any planned EBSs from the CollegeAIM matrix, and their delivery modality (e.g., group/individual, virtual/in-person). Data extraction from this review will provide baseline information about how many and which CollegeAIM strategies are planned for implementation, as well as provide data about trends (e.g., most institutions are using strategies focused on reducing binge drinking), and any potential areas of common adaptation (e.g., administering an in-person strategy online).

### Quantitative surveys

Following the strategic plan review, stakeholders will be invited via purposive and snowball sampling techniques to participate in an online survey assessing (1) demographic variables, (2) CollegeAIM (use, implementation outcomes (e.g., feasibility, acceptability)), and (3) perceptions of chosen prevention and intervention strategies, including EBSs (i.e., appropriateness, feasibility, sustainability) that serve as indicators of effective CollegeAIM use. EPIS constructs pertaining to outer context (factors impacting sustainability), inner context (organizational factors, e.g., implementation climate), and innovation (EBS fit; innovation complexity, cost, etc.) will be assessed to identify barriers and facilitators of CollegeAIM use and EBS selection. All measures are listed in the Appendix. We expect that approximately 220 stakeholders will complete the survey. All participants will re-consent for this portion of the study and will be compensated $20 for their time.

### Semi-structured interviews

Interviewees will be recruited from colleges that (1) wholly use, (2) partially adopt, and (3) deny use of CollegeAIM to select prevention and intervention strategies (also see the “Data analysis” section). We will recruit 5 stakeholders from each college to participate in the interview phase, or until saturation of themes is reached. Regardless of participation in prior phases, participants will be recruited to review and submit informed consent for the interview portion. We anticipate approximately 100–115 individuals will participate in interviews (approximately 4–5 per institution). Interviews will evaluate and explore stakeholders’ (a) perceptions and use of CollegeAIM, including use of specific steps such as strategic planning, reviewing annual student data from their respective institution, (b) effectiveness of CollegeAIM (i.e., selection of EBSs that fit the needs of their institution and are viewed as acceptable, appropriate, feasible, and sustainable) via examination of the prevention and intervention strategy selection process, and (c) key facilitators and barriers of CollegeAIM use, organized in the EPIS framework. All interviews will be audio recorded, transcribed, and coded for key themes by two independent coders. Discrepancies will be evaluated and resolved in consensus meetings with the coders and PI, and reliability will be reported. Participants will be compensated $40 for their participation in the interview.

### Focus groups

We will conduct six focus groups of approximately five participants each (*n* = 30), across all levels of CollegeAIM use (two focus groups with colleges that wholly use, two that partially use, and two that deny use of CollegeAIM). All participants in the focus groups will provide informed consent prior to participating. The focus groups will solicit feedback and discussion on the identified components (e.g., interactive training, ongoing consultation) of an organizational decision-support program for CollegeAIM, which will be derived from the data collected in earlier phases. Participants will be asked to provide their input on the collection of determinants and strategies for the program. All focus groups will be audio recorded, transcribed, and coded for key themes by two independent coders. Participants will be compensated $40 for their participation in the focus group.

#### Data analysis

##### Strategic plan review

Following data extraction, we will use descriptive statistics to summarize the inclusion of CollegeAIM in strategic plans, the number of planned EBSs per institution, as well as the number of EBSs with potential implementation adaptations. Finally, we will evaluate the proportion of strategies included in Strategic Plans that are listed in CollegeAIM (i.e., are most planned prevention and intervention strategies for alcohol evidence-based and listed in CollegeAIM?). Data extracted from the Strategic Plans will be used to recruit and group participants for later components (e.g., quantitative surveys, interviews), specifically by degree of CollegeAIM use at their respective institution.

##### Quantitative surveys

Descriptive statistics will be used to identify the number of institutions using CollegeAIM and categorize institutions into three groups: those that report wholly, partially, or not using CollegeAIM to select prevention and intervention strategies. This data will then inform sampling procedures for the interviews, so that institutions from each group are represented (also see the “Method” section). We will examine mean levels of acceptability, feasibility, and appropriateness ratings for the CollegeAIM tool and EBSs contained within CollegeAIM, to help identify potential barriers to CollegeAIM use. Using an ANOVA model, EPIS constructs will be evaluated across colleges that wholly, partially, or do not use CollegeAIM. Collectively, these results will provide information on CollegeAIM use, effective CollegeAIM use, and will provide a baseline set of potential barriers and facilitators to effective CollegeAIM use that will be explored further in the interview stage.

##### Interviews

Interviews will be coded by two independent coders for themes of CollegeAIM use, barriers and facilitators, and EPIS constructs. After consensus on specific coding decisions has been established, subsequent thematic analysis processes will be employed to identify themes relevant to the research questions. Themes and convergence across participants will point to components of effective CollegeAIM use and key barriers and areas for support, as identified by stakeholders. Theme differences across institutional characteristics (e.g., size) will also be examined.

Consistent with a sequential transformative design, the data will be integrated at the interpretation phase. At this phase, all available data will be integrated to describe how institutions are using CollegeAIM and identify common factors associated with “successful” CollegeAIM use and key barriers and facilitators to using CollegeAIM to select EBSs [20]. Themes (identified via thematic analysis) and quantitative survey results will be used to describe an integrated set of barriers and facilitators of CollegeAIM use with the EPIS constructs. Together, this information will provide data on key areas in which organizational support may be needed.

##### Focus groups

Data collected in the focus groups will be qualitative in nature and will center around feedback on the key components identified from data integration. Focus group transcripts will be coded by two independent coders, and themes will be identified via thematic analysis. Analyses will examine the frequency and convergence of responses across participants, in order to establish an understanding of the primary feedback and validation on the components for the support program.

## Discussion

### Innovation and impact

Addressing risky drinking among college students has been at the center of research, prevention, and clinical realms. With the advancement of available EBSs, there are a number of options available to higher education institutions trying to combat the problem of risky drinking. The College Alcohol Intervention Matrix (CollegeAIM) was developed by NIAAA to provide institutions with a vast amount of information on EBSs, within an organized and easy-to-follow tool. The goal of this tool is to help colleges in the selection of EBSs that fit their institutions. However, even with the availability of EBSs, we know that the path to implementation can be slow and wrought with a variety of challenges.

This protocol describes a project that takes an implementation science perspective to understand how institutions select and adopt prevention strategies and, more specifically, EBSs, for their campuses. Using a mixed-method design and in the context of the EPIS framework, we will investigate factors that help and hinder the use of CollegeAIM, as well as the general process in exploring and preparing for evidence-based alcohol preparation on their campuses. Evaluating the selection and adoption process specifically is essential, as we cannot expect extant EBSs to positively impact college students if they are not being implemented. Additionally, and perhaps more importantly, we need to understand what colleges need and what they are able to implement—and what factors influence this—so that we can better support institutions of higher education in providing alcohol and substance use prevention and intervention for their students. Perhaps colleges are not selecting EBSs because they do not know which ones exist, or maybe there are barriers within the context of the administration and/or financial sectors, or there may be colleges in which the programs and selected by non-specialists who are unfamiliar with the EBSs and/or implementation process. This mixed-method design provides us with a comprehensive understanding of the process from the stakeholders themselves. This will allow for an in-depth examination of the CollegeAIM tool and selection process of prevention and intervention strategies, and will gather rich information from the people making these decisions. As a result, we aim to identify components of a decision-support program that can aid colleges in this process.

### Dissemination plan

Results of this study will be disseminated via (1) submission of manuscripts to empirical journals, (2) professional presentations at national conferences, and (3) to stakeholder groups (e.g., college campus prevention experts).

## Data Availability

Data will be submitted to the National Institute on Alcohol Abuse and Alcoholism Data Archive (NIAAA_DA_). The datasets used and/or analyzed during the current study are available from the corresponding author on reasonable request.

## Appendix

### Measures

Measures focused on Current Suite Evidence-based strategies (those currently implemented at institution)

- Acceptability of Intervention Measure (AIM) [21] (4 items)^a^
- Intervention Appropriateness Measure (IAM) [21] (4 items)^a^
- Feasibility of Intervention Measure (FIM) [21] (4 items)^a^
- Selection of Strategies (3 items)^b^

Measures focused on College Alcohol Intervention Matrix (CollegeAIM) tool

- Acceptability of Intervention Measure (AIM) [21] (4 items)^a^
- Intervention Appropriateness Measure (IAM) [21] (4 items)^a^
- Feasibility of Intervention Measure (FIM) [21] (4 items)^a^
- Current Assessment of CollegeAIM Familiarity and Use (10 items)^b^
- Perceptions of CollegeAIM and CollegeAIM Resources (12 items)^b^

Measures focused on Evidence-based Strategies contained in the College Alcohol Intervention Matrix (CollegeAIM) tool

- Acceptability of Intervention Measure (AIM) [21] (4 items)^a^
- Intervention Appropriateness Measure (IAM) [21] (4 items)^a^
- Feasibility of Intervention Measure (FIM) [21] (4 items)^a^

Exploration, Preparation, Implementation, and Sustainment (EPIS) Focused Measures

- Implementation Climate Scale (ICS) [22] (18 items)
- Implementation Leadership Scale (ILS) [23] (12 items)
- Organizational Readiness for Implementing Change (ORIC) [24] (12 items)
- Program Sustainability Assessment Tool (PSAT) [25] (40 items)
- Evidence-based Practice Attitudes Scale (EBPAS) [26] (15 items)
- Evaluation of Support Tools for the Selection of Evidence-Based Strategies and CollegeAIM (9 items)^b^

*Notes*.

^a^Measures adapted for the “intervention” of interest (e.g., evaluating the acceptability of the “suite of strategies” currently implemented on their campus; evaluating the acceptability of the CollegeAIM tool)

^b^Given the specificity of the items (i.e., items assessing CollegeAIM) and lack of existing measures in this area, items were developed for this study.
